# Characterization and Comparative Analyses of Nuclear Mitochondrial DNAs in Genomes of the Leaf-Roller Moths (Lepidoptera: Tortricidae)

**DOI:** 10.3390/biology15060517

**Published:** 2026-03-23

**Authors:** Weifeng Peng, Jiayi Yu, Zhengbing Wang, Zhen Li, Xin Miao, Jin Liu, Jiahui Zhang, Liuyong Xie, Weili Ding, Keshi Ma, Mingsheng Yang

**Affiliations:** 1 College of Life Science and Agronomy, and Field Observation and Research Station of Green Agriculture in Dancheng County, Zhoukou Normal University, Zhoukou 466001, China; pengwf226@163.com (W.P.); 19636418028@163.com (J.Y.); wangzb191@163.com (Z.W.); lizhen920110@126.com (Z.L.); 15515675276@163.com (X.M.); 20242008@zknu.edu.cn (J.L.); zhangjiahui0806@163.com (J.Z.); xieliuyong10@163.com (L.X.); 2 Finance Office, Zhoukou Normal University, Zhoukou 466001, China; dingyi77@163.com

**Keywords:** DNA barcode, Lepidoptera, mitochondrial genome, mitochondrial pseudogenes, nuclear genome

## Abstract

During eukaryotes evolution, mitochondrial DNA (mtDNA) fragments integrate into nuclear genomes, forming nuclear mitochondrial DNA sequences (Numts). Tortricidae (Lepidoptera), a species-rich and economically critical family, lacks systematic characterization of Numts, which hinders reliable molecular research. Here, we systematically characterized Numts in 27 Tortricidae species spanning two subfamilies via genome download, mitochondrial genome annotation, and Numt identification and characterization. With each species’ mtDNA as query, Numt identification was performed with an E-value threshold of 10^−4^ and a sequence similarity cut-off of >60%, with a minimum length of 50 bp to exclude spurious hits. Results showed that all species contained Numts, with copy numbers varying drastically (9–208). Numt numbers positively correlated with nuclear genome length, but not mitochondrial genome length. Numts insertion flanking regions had significantly higher AT content than nuclear genome, indicating the insertion preference for AT-rich regions. Numts were predominantly derived from the mitochondrial *cox1* gene, highlighting the risk of co-amplification when *cox1* is used as a DNA barcode for species identification or phylogenetic studies. This study represents a systematic charaterizition of copy number, length distribution, insertion sequence preferences, and mitochondrial gene origins of Numts in Tortricidae, offering valuable references for refining molecular systematics, comparative genomics, and pest management in Tortricidae and related lepidopteran groups.

## Simple Summary

During eukaryotic evolution, mitochondrial DNA (mtDNA) fragments form Numts by integrating into nuclear genomes. Tortricidae lacked systematic Numt characterization, impeding molecular research. We analyzed Numts in 27 species (two subfamilies) with identification thresholds (E-value 10^−4^, >60% similarity, ≥50 bp). All species had 9–208 Numts, with counts correlating to nuclear genome length; Numts favored AT-rich insertion sites and derived mainly from *cox1*, highlighting risks for *cox1*-based molecular studies. This study systematically characterized Tortricidae Numt traits, informing molecular systematics and pest management for Tortricidae.

## 1. Introduction

Mitochondria are pivotal organelles for energy metabolism in eukaryotic cells, possessing independent mitochondrial DNA (mtDNA) [1]. According to the endosymbiotic theory, mitochondria evolved from bacteria engulfed by ancient eukaryotes, and a large number of mitochondrial genes have been transferred to the nuclear genome during long-term symbiosis [2]. The transformation of mtDNA into the nuclear genome forms nuclear mitochondrial DNAs (NUMTs), which are believed to be mainly non-functional sequences and may disrupt nuclear functional genes or contribute beneficially to gene evolution [3,4,5].

Numts provide novel insights into genome evolution while posing critical challenges to molecular studies that rely on mitochondrial markers. Their high sequence homology with authentic mtDNA can cause erroneous co-amplification during PCR amplification with universal primers, distorting species identification, phylogenetic analysis, population genetics, and genetics research [6,7]. Notable examples include misassigned novel genetic lineages in the leatherback sea turtle *Dermochelys coriacea* (attributed to Numt misamplification) [8] and artifactual reports of paternal mitochondrial inheritance linked to large-fragment Numt transmission [9]. Numts are widely distributed across eukaryotes, with copy number, length, and genomic distribution varying drastically among taxa. In insects, Numt researchs have focused primarily on the orders Coleoptera (e.g., [10,11]), Hymenoptera (e.g., [12,13,14]), and Diptera (e.g., [15]). In contrast, for the Lepidoptera, the largest insect order next to the Coleoptera and with high ecological and economic importance, characterization of Numt remains limited and insufficient [16], representing a prominent gap in current insect Numt research.

The family Tortricidae (Lepidoptera), generally known as the leaf-roller moths, is one of the most species-rich insect families, with over 11,000 described species worldwide [17,18]. Most Tortricidae species are phytophagous, and many are major agricultural and forestry pests that cause severe damage to trees and crops, including the codling moth *Cydia pomonella* and the oriental fruit moth *Grapholita molesta* [19]. These pests species incur substantial economic losses globally, rendering molecular-based research (e.g., species barcoding, phylogenetic analysis, pest population genetics) indispensable for their effective monitoring and management. In recent years, high-throughput sequencing has facilitated the accumulation of an expanding collection of Tortricidae nuclear and mitochondrial genomes [20,21,22], providing unprecedented resources for comparative genomic studies. However, systematic research exploring the distribution and molecular characteristics of Numts in Tortricidae genomes remains lacking, which is a critical oversight, as unrecognized Numts would invalidate mtDNA-based species identification and sistematic research for this ecologically and economically vital family.

To address this gap, we systematically identified and characterized Numts in 27 Tortricidae species using bioinformatic approaches based on available nuclear and mitochondrial genome data. Our specific objectives are as follows: (1) quantify copy number of Numt and total length across Tortricidae species; (2) analyze correlations between Numts abundance and nuclear/mitochondrial genome sizes; (3) investigate sequence characteristics of Numts insertion sites in the nuclear genome; (4) determine the mitochondrial gene origins of Numts. This study elucidates a key aspect of Tortricidae genome evolution and provides critical insights for future molecular research on this important insect family.

## 2. Materials and Methods

### 2.1. Data Collection

A total of 27 species of the family Tortricidae were selected as sample materials, including 10 from the subfamily Tortricinae and 17 from Olethreutinae. The mitochondrial and nuclear genome sequences of these species were retrieved from the GenBank database, https://www.ncbi.nlm.nih.gov/genbank/, on 15 August 2024. All 27 genomes analyzed were assembled at the chromosome level, with contig N50 values ranging from 0.105 Mb to 25.6 Mb and sequencing coverage spanning 1× to 150×. The detailed accession number and genome statistics are provided in Table 1.

### 2.2. Mitochondrial Genome Annotation

Among the 27 analyzed species, three (*Choristoneura fumiferana*, *Grapholita molesta*, and *Leguminivora glycinivorella*) have pre-annotated mitochondrial genomes available in the GenBank database. For these three species, manual curation of gene boundaries and start/stop codons was performed using MEGA X version 10 [23]. In contrast, the remaining 24 species only have the full-length mitochondrial sequences without annotation information. Thus, de novo annotation was conducted for these 24 speceis using the MITOS2 webserver [24] with the invertebrate genetic code. The gene boundaries of each mitochondrial gene were further validated via sequence alignment with closely related Tortricidae species using MAFFT v7.490 (https://mafft.cbrc.jp/alignment/software/; accessed on 15 October 2024), a widely used tool for accurate multiple sequence alignment.

### 2.3. Numt Identification and Characterization

The 37 annotated mtDNA genes of each species were extracted using PhyloSuite v1.2.1 [25] and checked with MEGA X [23]. For Numt identification, each mtDNA reference sequence (individual genes + full mtDNA contigs) was individually subjected to local BLASTN alignment (-task blastn) [26] using BLASTN v2.14.0 (https://blast.ncbi.nlm.nih.gov/; accessed on 15 October 2024) against the corresponding nuclear genome sequence to retrieve homologous sequences. An E-value threshold of 10^−4^ was adopted, as recommended by Tsuji et al. [27], and widely applied in previous insect Numts studies [12,14,28]. To avoid missing evolutionarily ancient Numts and exclude spurious alignments, this study excluded retrieval results with a matched sequence length ≤ 50 bp, selected matches with sequence similarity > 60% while excluding those with 100% query coverage [28,29]. Detailed information on each Numt from the 27 species is shown in the Table S1.

Based on the genomic positions of identified Numts, 100 bp upstream and downstream sequences were extracted from the nuclear genome, and MEGA X [23] was used for GC/AT content analyses. Correlation analysis between the Numt number and the length of nuclear/mitochondrial genomes was performed using the Spearman method implemented in the “cor.test()” function in R software version 4.5.2 (http://www.R-project.org/, accessed on 10 November 2024). Statistical comparisons of AT content between the inserted flanking sequences and the nuclear genome were performed using one-tailed *t*-test implemented in Microsoft Excel (Microsoft Corporation, Redmond, WA, USA, 2019).

## 3. Results

### 3.1. Characterization of Start and Stop Codons in Protein-Coding Genes in Mitochondrial Genomes

**Figure 1:**
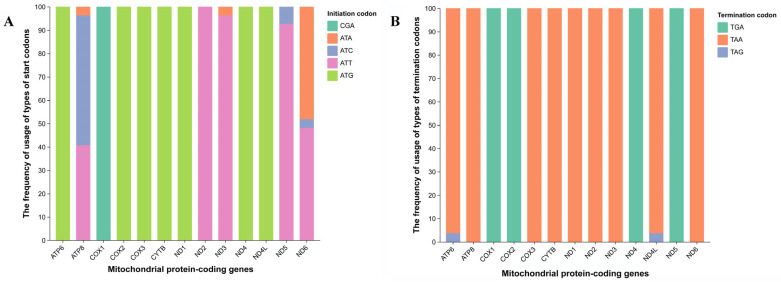
Relative use frequencies of start (**A**) and stop (**B**) codons of mitochondrial protein-coding genes.

The mitochondrial genomes of the 27 Tortricidae species ranged from 15,304 bp to 17,118 bp, with an average length of 16,172 bp. De novo annotation was performed for 24 species (the annotated mitochondrial genomes of *Choristoneura fumiferana*, *Grapholita molesta*, and *Leguminivora glycinivorella* are available on GenBank). All genomes contained the conserved 13 protein-coding genes (PCGs), 2 ribosomal RNA (rRNA) genes, and 22 transfer RNA (tRNA) genes. Five start codon types were identified (ATG, ATT, ATC, ATA, CGA; Figure 1A; Table S2), with ATG being the most frequent. In contrast to the diversity of start codons, only three stop codon types were detected (TAA, TAG, incomplete TGA; Figure 1B; Table S3), with TAA as the predominant form, which is consistent with general insect mitochondrial genome characteristics [17]. The relevance of mitochondrial codon usage to Numt origin lies in the shared codon characteristics between Numts and their parental mitochondrial DNA. This serves as a key link to trace Numt origin, verify their mitochondrial derivation, and distinguish Numts from native nuclear sequences.

### 3.2. Numts Content in the Nuclear Genome

Local BLASTN was employed to retrieve and screen Numts by aligning the nuclear genomes and mitochondrial genes of 27 Tortricidae species. The statistical results showed that the number of Numts varied from 9 to 208 across these species, with a mean count of 65. Specifically, *Pammene aurita* harbored the highest number of Numts (208), followed by *Hedya salicella* (207), *Epinotia demarniana* (172), *Epinotia bilunana* (153), *Notocelia uddmanniana* (94), and *Epinotia ramella* (89). In contrast, the lowest number of Numt (9 each) was detected in both *Tortricodes alternella* and *Acleris emargana* (Table 2). In terms of sequence length (Table 2), the total length of Numts differed among the Tortricidae species. However, the proportion of the total Numts length relative to the nuclear genome size ranged from 0.00022% to 0.01035%, with an average of 0.00269%. *Hedya salicella* exhibited the highest relative proportion (0.01035%), followed by *Epinotia demarniana* (0.00959%), *Pammene aurita* (0.00733%), *Cydia strobilella* (0.00710%), and *Epinotia bilunana* (0.00509%). By comparison, *Acleris emargana* had the smallest proportion (0.00022%). Based on the profiles of the Numts count and their relative length proportion in the nuclear genomes of the 27 Tortricidae species, it can be found that the total length of Numts only accounts for a tiny fraction of the nuclear genome in each species. Compared with the Numts data of fig wasps and bumblebees of the order Hymenoptera, the relative proportion of Numts in Tortricidae is lower, which is consistent with the statement that Hymenoptera may be a group rich in Numts [12,14].

### 3.3. Length Distribution of Numts

**Figure 2:**
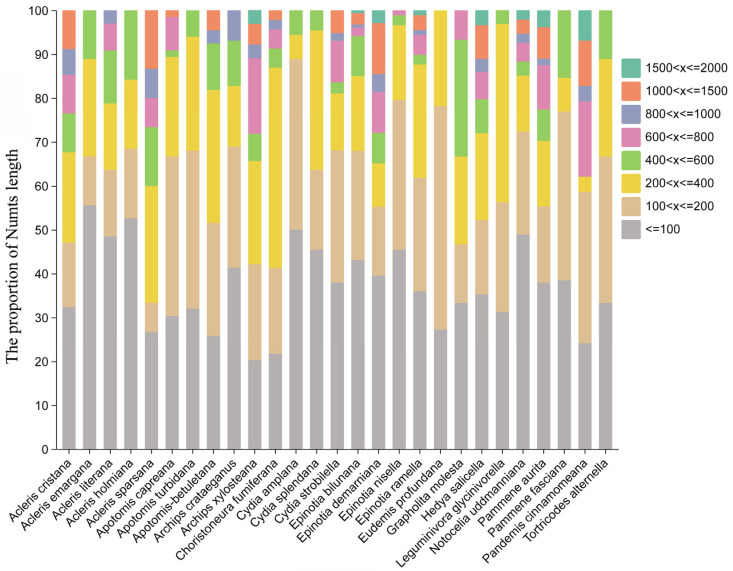
Numts length distribution of 27 Tortricidae species.

The Numts of 27 Tortricidae species exhibited a broad length distribution (Figure 2; Table S4), with distinct interspecific variations in their length profiles. Although the majority of Numts in most Tortricidae species were no longer than 200 bp, longer Numts exceeding 1500 bp were detected in several species, including *Archips xylosteana*, *Epinotia bilunana*, *Epinotia demarniana*, *Hedya salicella*, *Notocelia uddmanniana*, *Pammene aurita*, and *Pandemis cinnamomeana*.

### 3.4. Correlation Analysis Between Numts Abundance and the Lengths of Nuclear and Mitochondrial Genomes

**Figure 3:**
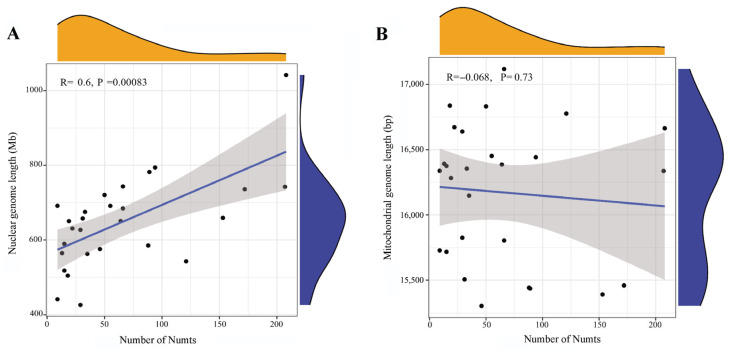
Correlation between the number of Numts and the lengths of the nuclear (**A**) and mitochondrial (**B**) genomes. The blue regression lines indicate the median value, and the wide gray shading indicates the 95% confidence interval. The marginal density plots (top/right) show the data distribution for the number of Numts.

Correlation analysis revealed a significant positive correlation between Numt abundance and nuclear genome length (R = 0.6, *p* = 0.00083; Figure 3A; Table S5), a finding inconsistent with observations in bumblebees [12]. This indicates nuclear genome length may represent a contributing factor to Numts accumulation in Tortricidae, likely because larger genomes typically contain more non-coding regions or repetitive sequences, which provide increased opportunities for mtDNA transfer and insertion events, thereby facilitating the accumulation and retention of Numts. In contrast, further investigation into the relationship between Numt abundance and mitochondrial genome length revealed no significant correlation (R = −0.068) with a high *p*-value of 0.73 (Figure 3B; Table S5), indicating that no statistically significant correlation existed between these two variables. This finding suggests that mitochondrial genome length may not exert a meaningful effect on Numt abundance in Tortricidae.

### 3.5. Sequence Preference of Numts Insertion

**Figure 4:**
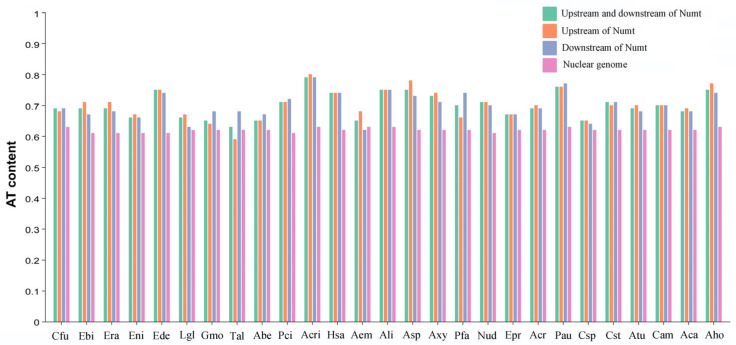
AT content of Numt flanking sequences and nuclear genomes of 27 Tortricidae species. Abe: *Apotomis betuletana*; Aca: *Apotomis capreana*; Aem: *Acleris emargana*; Aho: *Acleris holmiana*; Ali: *Acleris literana*; Asp: *Acleris sparsana*; Atu: *Apotomis turbidana*; Acr: *Archips crataeganus*; Acri: *Acleris cristana*; Axy: *Archips xylosteana*; Cam: *Cydia amplana*; Cfu: *Choristoneura fumiferana*; Csp: *Cydia splendana*; Cst: *Cydia strobilella*; Ebi: *Epinotia bilunana*; Ede: *Epinotia demarniana*; Eni: *Epinotia nisella*; Epr: *Eudemis profundana*; Era: *Epinotia ramella*; Hsa: *Hedya salicella*; Gmo: *Grapholita molesta*; Pau: *Pammene aurita*; Pci: *Pandemis cinnamomeana*; Pfa: *Pammene fasciana*; Lgl: *Leguminivora glycinivorella*; Nud: *Notocelia uddmanniana*; Tal: *Tortricodes alternella*.

To explore the sequence preferences associated with Numt insertion, we extracted and analyzed the composition of the 100 bp flanking sequences of Numts from 27 Tortricidae species. Our findings revealed that the AT content of the total Numt flanking sequences (including both upstream and downstream regions) was significantly higher than that of the nuclear genome (*p* < 0.01) (Figure 4; Table S6). This indicates that Numts tend to insert into AT-rich regions. Namely, the insertions of Numts in these Tortricidae insects exhibit a distinct AT preference. This pattern has also been reported in the genomes of Hymenopteran insects, including fig wasps and bumblebees [12,14]. In contrast, most Numts in the nuclear genome of vertebrates (such as pigs) are predominantly located in GC-rich regions [27]. In addition, the AT content of the upstream flanking sequences of Numts was comparable to that of the downstream flanking sequences (*p* = 0.23), which is not consistent with that of the bumblebees [12]. The latter reported that the AT content in upstream flanking sequences of Numts was consistently higher than that in the downstream flanking sequences.

### 3.6. Differential Distribution of Transfer Frequencies of Mitochondrial Protein-Coding Genes to the Nucleus

**Figure 5:**
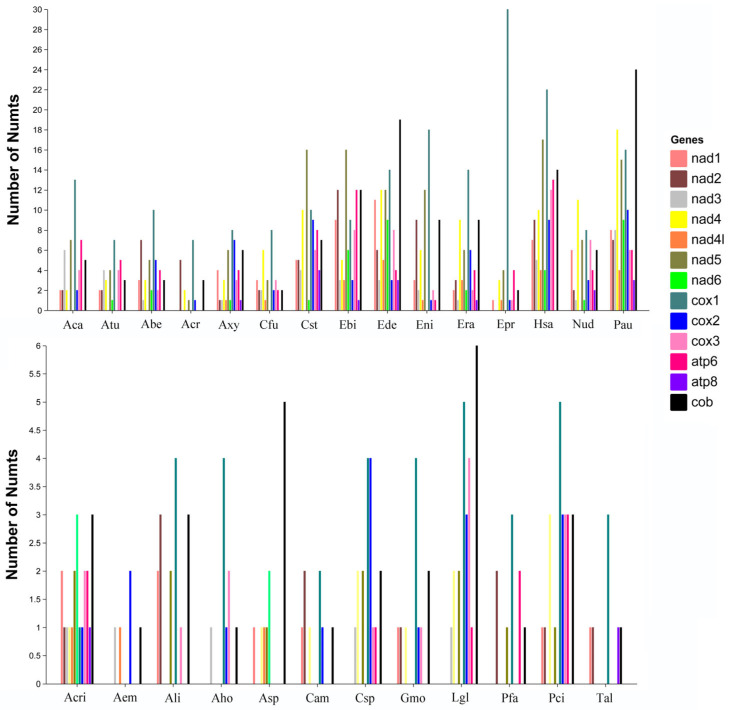
Number of Numts derived from 13 mitochondrial protein-coding genes of 27 Tortricidae species. Abe: *Apotomis betuletana*; Aca: *Apotomis capreana*; Aem: *Acleris emargana*; Aho: *Acleris holmiana*; Ali: *Acleris literana*; Asp: *Acleris sparsana*; Atu: *Apotomis turbidana*; Acr: *Archips crataeganus*; Acri: *Acleris cristana*; Axy: *Archips xylosteana*; Cam: *Cydia amplana*; Cfu: *Choristoneura fumiferana*; Csp: *Cydia splendana*; Cst: *Cydia strobilella*; Ebi: *Epinotia bilunana*; Ede: *Epinotia demarniana*; Eni: *Epinotia nisella*; Epr: *Eudemis profundana*; Era: *Epinotia ramella*; Hsa: *Hedya salicella*; Gmo: *Grapholita molesta*; Pau: *Pammene aurita*; Pci: *Pandemis cinnamomeana*; Pfa: *Pammene fasciana*; Lgl: *Leguminivora glycinivorella*; Nud: *Notocelia uddmanniana*; Tal: *Tortricodes alternella*.

To clarify the mitochondrial gene sequence origins of Numts, we quantified the number of Numts derived from each of the 13 mitochondrial protein-coding genes across all studied species. The results revealed substantial variation in the transfer frequencies of different protein-coding genes to the nuclear genome (Figure 5; Table S7). Among these mitochondrial genes, Numts originating from the *cox1* gene were the most abundant in the nuclear genome, followed by those derived from *cob* and *nad5*. In contrast, the *atp8* gene exhibited the lowest transfer frequency. Notably, the high abundance of *cox1*-derived Numts in Tortricidae species is consistent with previous observations in Hymenoptera taxa (including bumblebees and honey bees) [12,26] as well as in yeast [28], suggesting a potentially conserved pattern of preferential *cox1* gene transfer to the nucleus across diverse organisms.

## 4. Discussion

In this study, we characterized the quantity, length, origin, and insertion characteristics of the Numts via homology alignment between mitochondrial genes and nuclear genomes from 27 Tortricidae species. To our knowledge, this study represents the first detailed and systematic investigation of Numts in Tortricidae at the genomic scale, providing key empirical data and theoretical references for comparative genomics and molecular systematics of this family and related groups.

During the long-term evolutionary process of organisms, the transfer of mtDNA to the nucleus has been well-documented [30,31,32], and this transfer event is presumably an ongoing evolutionary process [33]. Correspondingly, Numts have been widely reported across a diverse range of organisms, including insects, pigs, and yeast [28,34,35]. However, Numt abundance varies substantially among distinct taxonomic groups. In insects, Hymenoptera species are recognized to possess higher Numt contents compared to other insect orders [14,36]. Notably, such significant interspecific variation in Numt abundance even exists within a taxonomic family [37] or lineage of closely related species [12]. Consistent with this pattern, the 27 Tortricidae species analyzed in the present study exhibited substantial differences in Numt counts (9–208), reflecting species- or lineage-specific evolutionary trajectories. As Numts are generally integrated into the nuclear genome during the repair of double-strand breaks (DSBs), previous studies have suggested that larger genomes, presumably harboring more DSBs, would contain more Numts [3,12,38,39]. Our analyses of Tortricidae species revealed a significant positive correlation between Numt number and nuclear genome size, aligning with findings in mosquitoes [15] but contrasting with those in fig wasps and bumblebees [12,14]. These discrepancies indicate that genome size is not the sole determinant of Numt abundance. Additional factors such as Numt loss rates, transposable element (TE) activity, and phylogenetic history may also modulate the transfer frequency of mitochondrial genes [14,40]. Alternatively, as summarized by Ding et al. [15], Numts analyses based on sufficient sampling sizes are more likely to stably reveal the intrinsic relationship between Numt content and genome size.

The preference for AT-rich regions as insertion sites of Numts is a prominent phenomenon in insect genomes, shaped by genomic context and evolutionary constraints. This preference is likely driven by the increased susceptibility of AT-rich regions to DSBs and subsequent non-homologous end-joining (NHEJ) repair [38], creating genomic “hotspots” for mtDNA integration. In bumblebees (Hymenoptera), a consistent AT preference is observed, with Numt flanking sequences (both upstream and downstream) exhibiting significantly higher AT content than the overall nuclear genome, and upstream regions showing greater AT enrichment than downstream ones [12]. This pattern aligns with findings in fig wasps (Hymenoptera), where Numts tend to insert into AT-rich genomic segments [14]. Similarly, in Orthoptera, Numts are frequently associated with transposable element (TE)-rich regions that are typically AT-biased, and TE activity-induced DSBs further facilitate Numt integration into these AT-rich loci [40]. For Tortricidae species, our analyses of 27 taxa confirm that AT-rich sequences are favored insertion sites. In contrast, Chrysomelidae beetles show no strong correlation between Numt insertion sites and genome-wide AT content, but localized AT-rich regions near centromeric heterochromatin still attract Numt integration [37]. The conserved AT bias in most taxa highlights the role of sequence composition in mediating mtDNA-nuclear transfer, as AT-rich regions reduce thermodynamic barriers for mtDNA fragment insertion and repair.

The predominance of *cox1*-derived Numts in Tortricidae is a striking finding, consistent with reports in other insect taxa such as bumblebees [12], mosquitoes [15], and Chrysomelidae beetles [37]. Mitochondrial *cox1* is the gold standard for DNA barcoding in insects, being used to identify species, resolve cryptic diversity, and construct phylogenetic trees at the species and population levels [41,42,43]. However, its high abundance in Numts means that phylogenetic analysis relying on universal *cox1* primers without rigorous Numt validation risk incorporating pseudogene sequences, leading to erroneous topological inferences. This is particularly critical for Tortricidae, a family with diverse notorious agricultural and forestry pests that inflict severe damage on trees and crops. Therefore, accurate species identification that excludes the potential interference of *cox1*-derived Numts is of paramount importance. To address this, integrated approaches are necessary, such as designing Tortricidae-specific *cox1* primers to reduce Numt co-amplification [14], combining multi-locus markers, e.g., *cox1* plus other Numt-drived nuclear markers [37], and leveraging bioinformatics tools to filter Numts based on sequence integrity and codon usage bias [12]. Accurate identification free from *cox1* Numt interference not only ensures precise pest diagnosis but also provides a robust foundation for tracking pest spread, assessing population dynamics, and formulating targeted control strategies to mitigate agricultural and forestry losses.

From a phylogenetic perspective, the existence of Numts represnt a conserved evolutionary process across organisms [30,31,32,33]. The variation in Numt accumulation may represent a phylogenetically informative trait for exploring phylogeny among different groups or species [40]. The correlation between phylogeny and Numts (nuclear mitochondrial DNA sequences) is manifested in both conserved patterns and lineage-specific divergences, making Numts valuable “molecular fossils” for inferring evolutionary relationships and historical events. Across insect taxa, Numt characteristics often align with phylogenetic clades. For example, in Schistocerca (Orthoptera), synaponumts (Numts shared by descendant species) are retained in the nuclear genomes of six congeneric species, reflecting integration events in their common ancestor and supporting the monophyly of the genus [40]. Similarly, in Chrysomelidae beetles, Numt distribution and abundance show phylogenetic clustering, with closely related species sharing similar Numt profiles (e.g., *atp8*-Numts as the least abundant type) [37]. In Tortricidae, compared with existing phylogenetic results based on mitochondrial genomes [17], we found that the phylogenetic relationships among the major groups within Tortricinae showed no significant positive correlation with the copy number (9–208) and length of Numts, a pattern consistent with that observed in bumblebees [12]. However, this finding requires further confirmation based on ample sampling from the speciose Tortricinae in future rearch. On the other hand, with over 11,000 recognized species, Tortricidae still has incompletely resolved phylogenetic relationships among higher groups and even congeneric species [17,22]. The universal presence of nuclear mitochondrial pseudogenes (Numts) highlights the necessity of rigorous validation of the current mtDNA-based phylogenetic results based on nuclear data, and Numt-based phylogenies complement mitochondrial data in this group in future research [37,40].

In future research, further validation could integrate long-read sequencing (e.g., PacBio/ONT) to resolve potential Numt fragmentation and contamination in short-read assemblies [44]. Experimental validation via PCR with Numt-specific primers and Sanger sequencing can confirm authentic insertions, while RNA-seq data will exclude expressed mtDNA misclassified as Numts [40]. These strategies will enhance the accuracy of Numt characterization in Tortricidae and refine molecular research reliability.

## 5. Conclusions

This study systematically characterizes Numts in 27 Tortricidae species spanning two subfamilies, uncovering their species-specific abundance (9–208 copies) origins of mtDNA. Numt number exhibits a significant positive correlation with nuclear genome length but not with mitochondrial genome length. In addition, the insertion sites of Numts show a significant AT preference. A novel conceptual insight is the conserved dominance of *cox1*-derived Numts, which is consistent across Tortricidae and supporting preferential mitochondrial gene transfer to the nucleus. These findings directly inform practical molecular research, such as *cox1*-based barcoding/phylogenetics for Tortricidae must account for Numt co-amplification risks. Future implications include optimizing pest identification via taxon-specific primers and multi-locus markers, and leveraging Numt traits to resolve Tortricidae phylogenies. This work advances comparative genomics and provides a scientific basis for sustainable pest management in Tortricidae and related groups.

## Figures and Tables

**Table 1:** The study species and genomic sources.

Subfamily	Species	Mitochondrial Genome Accession Number	Nuclear Genome Accession Number	Genome Contig N50	Genome Coverage
**Tortricinae**	*Acleris cristana*	OX411802	GCA_948252455.1	6.5 Mb	37×
	*Acleris emargana*	OV656865	GCA_927399475.2	16.7 Mb	25×
	*Acleris literana*	OX335698	GCA_946894065.1	20.4 Mb	29×
	*Acleris holmiana*	OX438793	GCA_949316455.1	1.1 Mb	40×
	*Acleris sparsana*	OV281315	GCA_923062465.1	19 Mb	37×
	*Archips xylosteana*	OX387374	GCA_947563465.1	20.4 Mb	41×
	*Choristoneura fumiferana*	NC_037395	GCA_025370935.1	0.105 Mb	1×
	*Cydia amplana*	OX419682	GCA_948474715.1	8.7 Mb	62×
	*Pandemis cinnamomeana*	OW028732	GCA_932294345.1	11.7 Mb	34×
	*Tortricodes alternella*	OX401990	GCA_947859335.1	10 Mb	58×
**Olethreutinae**	*Apotomis betuletana*	OW026326	GCA_932273695.1	12.4 Mb	34×
	*Apotomis capreana*	OX392526	GCA_947623375.1	5 Mb	31×
	*Apotomis turbidana*	LR990308	GCA_905147355.2	13.8 Mb	32×
	*Archips crataeganus*	OX402076	GCA_947859365.1	8.2 Mb	36×
	*Cydia splendana*	OU342899	GCA_910591565.2	6.5 Mb	36×
	*Cydia strobilella*	OX387703	GCA_947568885.1	10.6 Mb	36×
	*Epinotia bilunana*	OX346280	GCA_947049275.1	8.3 Mb	28×
	*Epinotia demarniana*	OX244287	GCA_945867215.1	13.5 Mb	30×
	*Epinotia nisella*	OW028701	GCA_932294315.1	21.2 Mb	39×
	*Epinotia ramella*	OX388228	GCA_947578815.1	14 Mb	31×
	*Eudemis profundana*	OX344822	GCA_947034925.1	16.6 Mb	37×
	*Grapholita molesta*	HQ116416	GCA_022674325.2	18.2 Mb	150×
	*Hedya salicella*	FR990121	GCA_905404275.2	25.6 Mb	25×
	*Leguminivora glycinivorella*	MH013481	GCA_023078275.1	4.2 Mb	85.48×
	*Notocelia uddmanniana*	LR991080	GCA_905163555.1	6.7 Mb	18×
	*Pammene aurita*	OX352317	GCA_947086415.1	9.2 Mb	24×
	*Pammene fasciana*	OU452300	GCA_911728535.1	16 Mb	36×

**Table 2:** The content of Numts in the nuclear genomes of the 27 Tortricidae species.

Species	Number of Numt	Length of Numts (bp)	The Percentage of Numt Length to the Nuclear Genome (%)
*Acleris cristana*	34	13,146	0.00234
*Acleris emargana*	9	1500	0.00022
*Acleris holmiana*	19	3632	0.00056
*Acleris literana*	33	7563	0.00112
*Acleris sparsana*	15	6386	0.00108
*Apotomis betuletana*	66	18,523	0.00271
*Apotomis capreana*	66	14,391	0.00194
*Apotomis turbidana*	50	8761	0.00122
*Archips crataeganus*	29	6899	0.00110
*Archips xylosteana*	64	26,076	0.00401
*Choristoneura fumiferana*	46	4157	0.00072
*Cydia amplana*	18	2246	0.00045
*Cydia splendana*	22	3612	0.00057
*Cydia strobilella*	121	38,506	0.00710
*Epinotia bilunana*	153	33,531	0.00509
*Epinotia demarniana*	172	70,532	0.00959
*Epinotia nisella*	88	12,819	0.00219
*Epinotia ramella*	89	22,787	0.00291
*Eudemis profundana*	55	9557	0.00138
*Grapholita molesta*	15	4261	0.00082
*Hedya salicella*	207	76,799	0.01035
*Leguminivora glycinivorella*	32	5993	0.00090
*Notocelia uddmanniana*	94	23,444	0.00295
*Pammene aurita*	208	76,405	0.00733
*Pammene fasciana*	13	2068	0.00037
*Pandemis cinnamomeana*	29	14,033	0.00329
*Tortricodes alternella*	9	1808	0.00041

## Data Availability

The data presented in this study are included in the Supplementary Material. Further inquiries can be directed to the corresponding authors.

## Supplementary Materials

The following supporting information can be downloaded at https://www.mdpi.com/article/10.3390/biology15060517/s1. Table S1: The information on Numts from 27 Tortricidae species; Table S2: The frequency of initiation codon usage in mitochondrial protein-coding genes of the 27 Tortricidae species; Table S3: The frequency of termination codon usage in mitochondrial protein-coding genes of the 27 Tortricidae species; Table S4: Length distribution of the Numts from the 27 Tortricidae species; Table S5: The content of Numts in the nuclear genomes of the 27 Tortricidae species; Table S6: AT content of Numts flanking sequences and nuclear genomes; Table S7: The number of Numts derived from 13 mitochondrial protein-coding genes.
